# Mitochondrial oxidative stress, mitochondrial ROS storms in long COVID pathogenesis

**DOI:** 10.3389/fimmu.2023.1275001

**Published:** 2023-12-22

**Authors:** Kunwadee Noonong, Moragot Chatatikun, Sirirat Surinkaew, Manas Kotepui, Rahni Hossain, Kingkan Bunluepuech, Chanittha Noothong, Aman Tedasen, Wiyada Kwanhian Klangbud, Motoki Imai, Fumitaka Kawakami, Makoto Kubo, Yoshimasa Kitagawa, Hiroshi Ichikawa, Takuro Kanekura, Suriyan Sukati, Voravuth Somsak, Lunla Udomwech, Takafumi Ichikawa, Veeranoot Nissapatorn, Jitbanjong Tangpong, Hiroko P. Indo, Hideyuki J. Majima

**Affiliations:** ^1^ School of Allied Health Sciences, Walailak University, Nakhon Si Thammarat, Thailand; ^2^ Research Excellence Center for Innovation and Health Products (RECIHP), School of Allied Health Sciences, Walailak University, Nakhon Si Thammarat, Thailand; ^3^ Center of Excellence Research for Melioidosis and Microorganisms, Walailak University, Nakhon Si Thammarat, Thailand; ^4^ School of Medicine, Walailak University, Nakhon Si Thammarat, Thailand; ^5^ Department of Molecular Diagnostics, School of Allied Health Sciences, Kitasato University, Sagamihara, Japan; ^6^ Regenerative Medicine and Cell Design Research Facility, School of Allied Health Sciences, Kitasato University, Sagamihara, Japan; ^7^ Department of Regulation Biochemistry, Kitasato University Graduate School of Medical Sciences, Sagamihara, Japan; ^8^ Department of Health Administration, School of Allied Health Sciences, Kitasato University, Sagamihara, Japan; ^9^ Division of Microbiology, School of Allied Health Sciences, Kitasato University, Sagamihara, Japan; ^10^ Department of Environmental Microbiology, Graduate School of Medical Sciences, Kitasato University, Sagamihara, Japan; ^11^ Oral Diagnosis and Medicine, Division of Oral Pathobiological Science, Graduate School of Dental Medicine, Hokkaido University, Sapporo, Japan; ^12^ Graduate School of Life and Medical Sciences, Doshisha University, Kyoto, Japan; ^13^ Department of Dermatology, Kagoshima University Graduate School of Medical and Dental Sciences, Kagoshima, Japan; ^14^ Department of Oncology, Kagoshima University Graduate School of Medical and Dental Sciences, Kagoshima, Japan; ^15^ Amanogawa Galaxy Astronomy Research Center, Kagoshima University Graduate School of Engineering, Kagoshima, Japan

**Keywords:** COVID-19, oxidative stress, mitochondrial ROS storms, long Covid, mitochondria

## Abstract

**Significance:**

This review discusses the coronavirus disease 2019 (COVID-19) pathophysiology in the context of diabetes and intracellular reactions by COVID-19, including mitochondrial oxidative stress storms, mitochondrial ROS storms, and long COVID.

**Recent advances:**

The long COVID is suffered in ~10% of the COVID-19 patients. Even the virus does not exist, the patients suffer the long COVID for even over a year, This disease could be a mitochondria dysregulation disease.

**Critical issues:**

Patients who recover from COVID-19 can develop new or persistent symptoms of multi-organ complications lasting weeks or months, called long COVID. The underlying mechanisms involved in the long COVID is still unclear. Once the symptoms of long COVID persist, they cause significant damage, leading to numerous, persistent symptoms.

**Future directions:**

A comprehensive map of the stages and pathogenetic mechanisms related to long COVID and effective drugs to treat and prevent it are required, which will aid the development of future long COVID treatments and symptom relief.

## 1 Introduction

Coronavirus disease 2019 (COVID-19) was first reported in Wuhan, China, in late December 2019. After the emergence of SARS-CoV-2 infections in December 2019 (1), the detailed symptoms were introduced in February 2020 by Huan et al. (2), Chen et al. (3), and Chan et al. (4). Lu et al. (5) concluded that the 2019-nCoV was a new human-infecting beta-coronavirus, sufficiently differing from SARS coronavirus (SARS-CoV). The COVID-19 pandemic has spread in the whole world. Yang et al. (6) compared the time course of the 2003 SARS pandemic and the 2020 novel coronavirus epidemic in China; the two diseases followed a similar course of events, although the number of cases was relatively limited in China during the 2003 SARS pandemic–the pneumonia outbreak associated with a new coronavirus of probable bat origin (7). SARS-CoV 2, Pangolin-CoV, SARS-CoV, Middle East respiratory syndrome CoV, and Bat-CoV viruses evolve quickly (8, 9). The first symptom reported for COVID-19 was pneumonia (10). This represents COVID-19 infection started from a respiratory tract infection that included fever, dizziness, and cough (11). Several variants of COVID-19, namely Alpha, Beta, Gamma (12), Delta (12–14), and Omicron (15, 16), have resulted in subsequent outbreaks in many countries worldwide. Despite improvements in the management of COVID-19, severe infection cases and COVID-19-related fatalities still occur. Presumed hospital-related transmission of COVID-19 was suspected in 41% of patients, 26% of patients received ICU care, and mortality was 4.3% (17). In addition, there is substantial concern regarding a complication known as long COVID-19.

Sudreres et al. (18) analyzed data from 4,182 COVID-19 cases and reported that the number of long COVID-19 cases was 558 (13.3%) participants reporting symptoms lasting ≥28 days, 189 (4.5%) for ≥8 weeks, and 95 (2.3%) for ≥12 weeks. They also reported that long COVID is characterized by symptoms of fatigue, headache, dyspnea, and anosmia and is likely associated with factors such as increasing age, increasing body mass index, and female sex (18). Moreover, patients experiencing more than five symptoms during the first week of illness were more likely to experience long COVID (odds ratio = 3.53 [2.76–4.50]) (18). A recent report by the Center for Disease Control and Prevention (CDC) National Center for Health Statistics (19), announced that new data from the Household Pulse Survey indicate that more than 40% of adults in the United States reported having COVID-19 in the past. Nearly one in five of those (19%) are currently still having symptoms of long COVID. These findings highlight the importance of investigating the cause of long COVID and developing potential treatments.

In this review, we focus on long COVID, a pathophysiological condition characterized by the recurrence of symptoms weeks or months after traces of the COVID-19 virus disappear. Despite abundant data on long COVID, its underlying causes and effective treatments remain unknown. This review focuses on the underlying cause of long COVID and its occurrence, providing essential insights into understanding long COVID.

## 2 COVID-19 and diabetes mellitus

### 2.1 The influence of DM on COVID-19 infection

Following the declaration of COVID-19 as a worldwide pandemic, patients with COVID-19 and DM were more likely to develop severe or critical disease conditions with more complications. The results of the meta-analysis showed that DM seemed to contribute to an increased mortality risk among hospitalized patients with COVID-19 compared to those without DM (**Table 1**, **Supplementary Figure 1**) (20–26). Hussain et al. (20) reported a significantly higher risk of intensive care unit (ICU) admission in patients with COVID-19 and DM compared to those without DM, with a pooled risk ratio of 1.88 (1.20–2.93%), *p* < 0.006, as well a significantly higher mortality risk, with a pooled risk ratio of 1.61 (95% confidence interval: 1.16−2.25%), *p* = 0.005. Shang et al. (21) reported that these patients had higher and more severe COVID-19 infection rates than those without DM, at 21.4% and 10.6%, respectively (*p* < 0.01), and were associated with an increased mortality risk (28.5 vs. 13.3%, respectively; *p* < 0.01; odds ratio: 2.14). Based on the data in **Table 1**, an *in silico* analysis of the overall meta-analysis results was performed. The forest plot of the pooled case mortality ratio in patients with COVID-19 and DM is shown in **Supplementary Figure 1**. Among a total of 4,450,522 patients with COVID-19, the average mortality ratio for patients with DM was 1.67 on average (**Table 1**) (20–26). Moreover, hyperglycemia strongly predicts poor prognosis in patients with COVID-19 (27). Paul et al. (28) discussed the effects of oxidative stress management on alleviating COVID-19 symptoms in patients with DM as a comorbidity. It is considered that both COVID-19 and DM are oxidative diseases, so the patients receive oxidative stress synergistically.

**Figure 1:**
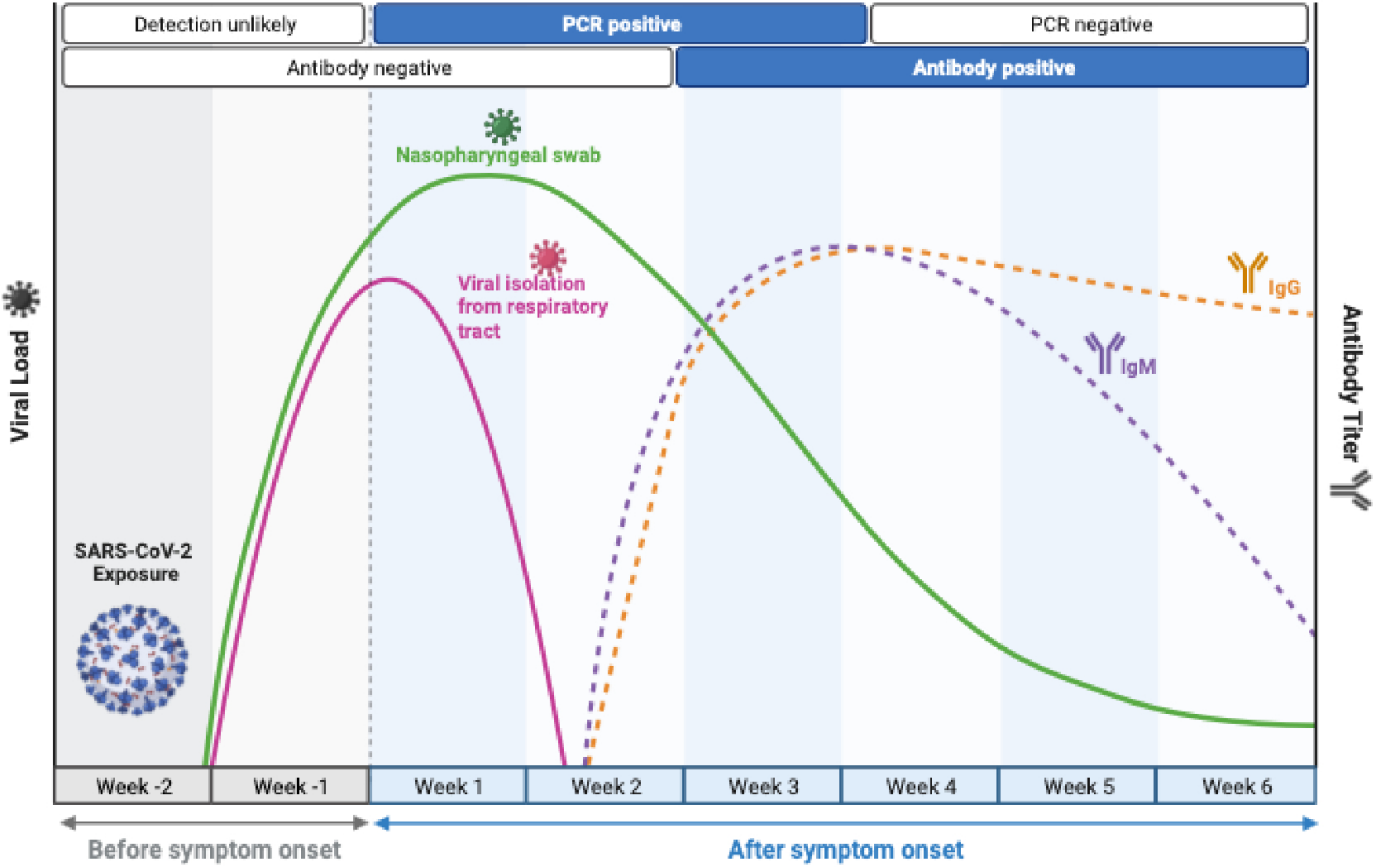
Timelines of long COVID. Long COVID-19 is defined as persistent symptoms and or delayed or long-term complications beyond 4 weeks from the onset of symptoms. (**Figure 1** adapted from BioRender).

**Table 1:** Total numbers of patients, infection rate, infection rate (DM), infection rate (COVID-19), intensive care unit (ICU) admission rate, case mortality ratio (DM vs. others) of patients with COVID-19 and diabetes mellitus (DM) in seven aricles (20–26) and the average 1.67.

Total no. of patients	Infection rate (DM)	Infection rate (COVID-19)	ICU admission rate ratio (DM vs. Others)	Case mortality ratio (MR;DM vs. others) 95% confidence interval (CI)	Reference
23,007 patients	15% (95% CI: 12−18%), *p* < 0.0001		1.88 (1.20−2.93), *p* = 0.006	Ratio: 1.61 (95% CI: 1.16−2.25), *p* = 0.005	Hussain et al., 2020 (20)
31,067 patients		DM 21.4%, Non-DM 10.6% (*p* < 0.01)		Ratio: 2.21 (95% CI: 1.83−2.66, I^2^ 50%), *p* < 0.01 (28.5% vs. 13.3%), *P* < 0.01	Shang et al., 2020 (21)
45,775 hospitalized COVID-19 patients	20% (95% CI: 15.0–25.0; I^2 =^ 99.3%)			Ratio: 1.82 (95% CI: 1.25−2.39), DM 20.0% (95% CI: 15.0–26.0; I^2^ 96.8%), Non-DM 11.0% (95% CI: 6.0–16.0; I^2^ 99.3%)	Saha et al., 2021 (22)
18,506 patients	20%			Ratio: 1.65 (95% CI 1.35−1.96; I^2^ 77.4%), *p* < 0.001	Palaiodimos et al., 2021 (23)
35,486 patients	17[15;19]%			5867 deaths (16.53%), Ratio: 1.85 (95% CI: 1.36−2.51) *p* < 0.01	Corona et al., 2021 (24)
25,934 patients	16.9% (n = 4381)			Ratio: 1.83 (95% CI: 1.61 - 2.05), (DM vs. Non-DM: 22.14% vs. 12.81%) *p* < 0.05	Gupta et al., 2021 (25)
4,270,747 COVID-19 patients and 43,203,759 controls.				Ratio: (risk ratio, 1.66; 95% CI 1.38; 2.00) *p* < 0.0001	Ssentonga et al., 2022 (26)
**Total** 4,450,522 patients		Average of DM patients with COVID-19: 18.9%		Average ratio of mortality ratio for DM patients: 1.67	

### 2.2 DM and long COVID

Steenblock et al. (29) suggested the increased risk for people with diabetes in the acute phase of COVID-19, and this patient group seemed to be more often affected by long COVID and experience more long-term consequences than people without diabetes. However, the mechanisms behind these discrepancies between people with and without diabetes concerning COVID-19 are not entirely understood yet (30). Furthermore, Xie et al. (31) examined the risk of diabetes following COVID-19 infection and described that In the post-acute phase of the disease (in long COVID phase). Xie and Al-Aly compared with the contemporary control group, people with COVID-19 exhibited an increased risk of incident diabetes (31). Rizvi et al. (32) suggested that COVID-19 virus directly attacks the beta cells of islets by binding with ACE2. The other factors of overactivated inflammation, such as elevation in neutrophils, IL-6, and CRP, and imbalanced immunoreaction, such as reduction in lymphocytes, monocytes, CD4+ and CD8+ T cells cause insulin insufficient synthesis and systemic insulin resistance. These situations cause impaired glucose regulation and new-onset diabetes (32). Bramante et al. (33) described that outpatient treatment with metformin reduced long COVID incidence by about 41%, with an absolute reduction of 4.1%, compared with placebo. Overall, 93 (8.3%) of 1126 participants reported receipt of a long COVID diagnosis by day 300. The cumulative incidence of long COVID by day 300 was 6.3% (95% CI 4·2-8·2) in participants who received metformin and 10.4% (7.8-12.9) in those who received identical metformin placebo (hazard ratio [HR] 0.59, 95% CI 0.39-0.89; *p*=0·012). Metformin has clinical benefits when used as outpatient treatment for COVID-19 and is globally available, low-cost, and safe.

V’kovski et al. (34) described an essential understanding of SARS-CoV-2 infection throughout the intracellular viral life cycle. Mitochondria could be involved in the intracellular viral life cycle.

## 3 Prognosis for severity of COVID-19

The prognosis of severe COVID-19 cases is essential. Rizzi et al. (35) summarized the most promising biomarkers to predict the severity of COVID-19. Those are IP10 (36), Gas6 (37–39), serum SARS-CoV-2 nucleic acid (RNAaemia) (40), and Calcitonin Gene-Related Peptide (CGRP) plasma levels (41). Chen et al. (40) described that RNAaemia is closely related to IL-6. Therefore, IL-6 could also be an essential factor in predicting the severity of COVID-19.

## 4 COVID-19 virus influences mitochondria of the infected patient

Jackson et al. explained the mechanisms of SARS-CoV-2 entry into host cells; the binding of the spike (S) protein to its receptor, angiotensin-converting enzyme 2 (ACE2), and subsequent membrane fusion (42). It is shown that an association with COVID-19 causes redox imbalance or oxidative stress (43, 44). Viral infections alter mitochondrial dynamics at various levels and impact mitochondrial functioning (45). Upon SARS-CoV-2 entry, the RNA genome is released, and translated, and the resulting structural and non-structural proteins interact with mitochondrial components. Then, SARS-CoV-2 escape from mitochondria-mediated innate immune response and establish its infection (46). SARS-CoV-2 may manipulate mitochondrial function indirectly, first by ACE2 regulation of mitochondrial function, and once it enters the host cell, open-reading frames (ORFs) such as ORF-9b can directly manipulate mitochondrial function to evade host cell immunity and facilitate virus replication and COVID-19 disease. Manipulation of host mitochondria by viral ORFs can release mitochondrial DNA (mtDNA) in the cytoplasm, activate mtDNA-induced inflammasome, and suppress innate and adaptive immunity (47). The viruses may induce mtDNA degradation, alter mitochondrial metabolic pathways, impact mitochondrial membrane potential, and modify the mitochondrial intracellular number and distribution, thereby influencing apoptosis, mitochondrial homeostasis, or evade mitochondrial antiviral signals (48–50). Ajaz et al. investigated functional mitochondrial changes in live peripheral blood mononuclear cells (PBMCs) from patients with COVID-19 and subsequent changes in the inflammatory pathways. They demonstrated mitochondrial dysfunction, metabolic alterations with an increase in glycolysis, and high levels of mitokine in PBMCs from patients with COVID-19. They found that levels of fibroblast growth factor 21 mitokine correlate with COVID-19 disease severity and mortality (51)..

Mitochondria appear to be important in COVID-19 pathogenesis because of its role in innate antiviral immunity, as well as inflammation (52). Mitochondrial antiviral signaling protein (MAVS) is an innate immune adaptor on the outer mitochondrial membrane that acts as a switch in the immune signal transduction response to viral infections. Increased aerobic glycolysis provides material and energy for viral replication upon viral infection. MAVS is the only protein specified downstream of retinoic acid-inducible gene I (RIG-I) that bridges the gap between antiviral immunity and glycolysis. MAVS binding to RIG-I inhibits MAVS binding to Hexokinase (HK2), thereby impairing glycolysis (53). In contrast, excess lactate production inhibits MAVS and the downstream antiviral immune response, facilitating viral replication (53, 54).

SARS-CoV-2 RNA enters macrophages, MAVS and mitofusin 1 and 2 causing mitochondrial dysfunction and the subsequent increase in ROS generation and mt-DNA into the cytosol. This causes the activation and recruitment of NLR family pyrin domain containing 3 (NLRP3). Wu et al. have reported that MAVS mediates NF-κB and type I interferon signaling during viral infection and is also required to activate the NLR family pyrin domain containing 3 (NLRP3) that triggers an immune response (55).. Apoptosis-associated speck-like protein containing a caspase recruitment domain (ASC) protein (56), and Caspase-1, which assemble to create the NLRP3 inflammasome (57). The activated NLRP3 inflammasome cleaves the cytokines Pro-IL-1B and Pro-IL-18 into their mature and biologically active forms (IL-1B and IL-18), thus exacerbating the inflammation state (58). Moreno Fernández-Ayala suggested that chronic inflammation caused by mitochondrial dysfunction is responsible for the explosive release of inflammatory cytokines causing severe pneumonia, multi-organ failure, and finally death in COVID-19 patients (59).

SARS-CoV-2 enters the cells, and the RNA and RNA transcripts capture the mitochondria, and disrupt the mitochondrial electron transport chain (60). Prasada Kabekkodu et al. suggested that SARS CoV proteins localize in the mitochondria, increase reactive oxygen species (ROS) levels, perturbation of Ca2+ signaling, changes in mtDNA copy number, mitochondrial membrane potential (MMP), mitochondrial mass, and induction of mitophagy (61). Guarnieri et al. suggested that after the COVID-19 virus infection, there was a systemic host response followed by viral suppression of mitochondrial gene transcription and followed by induction of glycolysis (62). Even when the virus was cleared, mitochondrial function in the heart, kidney, liver, and lymph nodes remained impaired, leading to severe COVID-19 pathology (62). Miller et al. reported that SARS-CoV-2 did not dramatically regulate (1) mtDNA-encoded gene expression or (2) MAVS expression, and (3) SARS-CoV-2 downregulated nuclear-encoded mitochondrial (NEM) genes related to cellular respiration and Complex I (63). Bhowal et al. reported that open reading frames (ORFs) of COVID-19, ORF-9b, and ORF-6 impair MAVS protein and suppress innate antiviral response activation (64).

Duan et al. found significant changes in mitochondrion-related gene expression, mitochondrial functions, and related metabolic pathways in patients with COVID-19, analyzing RNA-sequencing dataset of lung tissue and blood from COVID-19 patients (65). Yang et al. exhibited that SARS-CoV-2 membrane protein (M protein) could induce mitochondrial apoptosis pathway via B-cell lymphoma 2 (BCL-2) ovarian killer (BOK) without BAK and BAX, thus exacerbating SARS-CoV-2 associated lung injury in vivo (66).

## 5 Long COVID

### 5.1 Long COVID as a well-developed feature

COVID-19 is a significant pandemic resulting in substantial mortality and morbidity worldwide. Of the individuals affected, approximately 80% had mild-to-moderate disease, and among those with severe disease, 5% developed critical illness (67). A few of those who recovered from COVID-19 developed persistent or new symptoms lasting for weeks or months; this is called “long COVID,” “long haulers,” or “post-COVID syndrome” (68, 69). Nguyen et al. (70) reported the long-term persistence of dyspnea in patients with COVID-19.

Long COVID was defined by Crook et al. (71), and published on May 5, 2020, in BMJ Opinion, where he shared his experience of seven weeks on a “roller coaster of ill health” following COVID-19 (72). Long COVID is now recognized in the National Institute for Health and Care Excellence guidelines on managing the long-term effects of COVID-19 (73). Datta et al. (74) define patients with long COVID or long haulers as individuals with ongoing symptoms of COVID-19 that persist beyond four weeks from the initial infection.

### 5.2 Long COVID symptoms

Long COVID is a debilitating illness in at least 10% of severe SARS-CoV-2 infections (75). COVID-19 is now recognized as a multi-organ disease with a broad spectrum of manifestations. As for post-acute viral syndromes, there is an increasing number of reports of persistent and prolonged effects following acute COVID-19. There are currently no validated effective treatments for long COVID (75). Common symptoms of long COVID include fatigue, shortness of breath, cough, joint pain, chest pain, muscle aches, and headaches (76). Patient advocacy groups, many members of which identify as long haulers, have contributed to recognizing post-acute COVID-19 (77). In the absence of a virus in patients after COVID-19 infection, long COVID causes symptoms similar to those of myalgic encephalomyelitis/chronic fatigue syndrome (ME/CFS) (78–80). Linhoff et al. reviewed recent data on Long-COVID and Long-COVID-related fatigue (LCOF), focusing on cognitive fatigue (81). Regarding long COVID pathological co-factors, Bellan et al. (82) described that the condition of proinflammatory cytokines in patients can be essential. Explanations for “long COVID” include immune imbalance, incomplete viral clearance, and potentially even mitochondrial dysfunction (83). Of note, oxidative stress might be an underlying cause of long COVID (84).

### 5.3 Long COVID and mitochondria

Lactic acid, lactate/pyruvate ratio, ornithine/citrulline ratio, and arginine were identified as the most relevant metabolites for distinguishing long COVID patients even two years after acute COVID-19 infection (85). Long COVID causes mitochondrial dysfunction, redox state imbalance, impaired energy metabolism, and chronic immune dysregulation.

**Figure 2:**
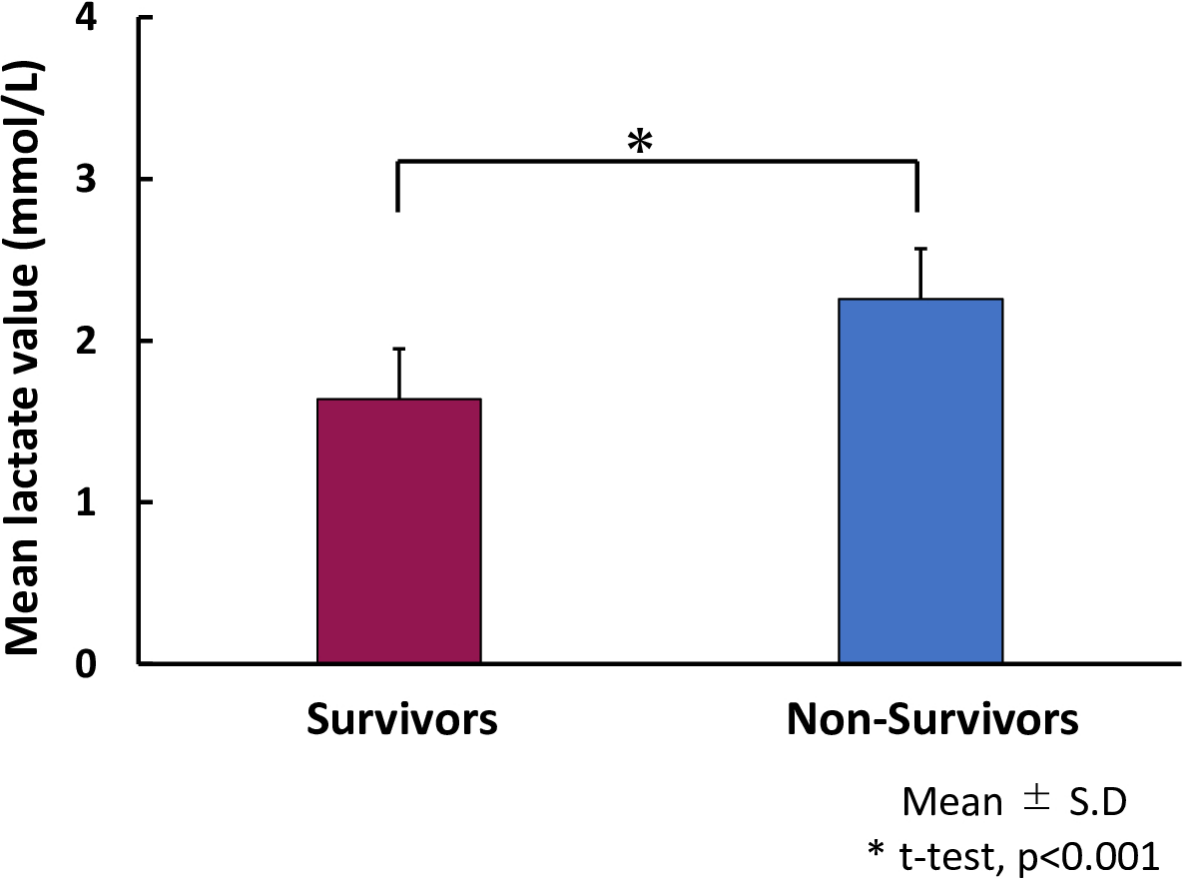
Lactate levels in coronavirus disease 2019 (COVID-19) survivors vs. non-survivors. Bar graph shows mean lactate levels in COVID-19 survivors (red bar) and non-survivors (blue bar). The blue dot is an outlier. Remended from Carpenè et al. (2021) (86) the and articles 87–96.

Carpenè et al. (86) demonstrated that blood lactate levels were higher in severe cases of non-survivor patients with COVID-19 than in non-severe survivor cases, as shown in **Supplementary Figure 2** (87–96). **Figure 2** shows the blood lactate levels in coronavirus disease 2019 (COVID-19) survivors vs. non-survivors taken from the results of references 87-96, suggesting that the blood lactate levels in COVID-19 non-survivors are significantly higher than the survivors. The results of **Supplementary Figure 2** show that in long COVID patients, intracellular energy production tends to use glycolysis rather than using mitochondrial oxidative phosphorylation. The meta-analysis showed that lactate dehydrogenase (LDH) was also increased in patients with COVID-19 and associated with relatively poor outcomes (97). Lactate dehydrogenase is markedly elevated in plasma and strongly associated with mortality in severe COVID-19 (98). This finding is consistent with the potential explanations for “long COVID,” which include mitochondrial dysfunction (83). Vitamin D is an immunomodulatory hormone with proven efficacy against various upper respiratory tract infections; it can inhibit hyperinflammatory reactions and accelerate the healing process in affected areas, especially lung tissue. Moreover, vitamin D deficiency is associated with the severity and mortality of COVID-19 cases, with a high prevalence of hypovitaminosis D found in patients with COVID-19 and acute respiratory failure (76). Antonelli et al. (99) described that among Omicron cases, 4.5% of people experienced long COVID, whereas 10.8% experienced long COVID following Delta variant infection. Hernández-Aceituno et al. (100) described that the ongoing symptomatic COVID (4–12 weeks), post-COVID-19 (> 12 weeks with symptoms), and long COVID cases were less frequent in Omicron cases, compared with Alpha or Delta cases. These findings suggest that patients infected with the Omicron variant are less likely to experience long COVID.

Antonelli et al. (99) also described that after infection with Omicron or Delta variants, less than three months after vaccination, the long COVID odds ratio increases compared to the “3 to 6 month” and “prior to 6-month” groups. Vaccinated individuals are occasionally diagnosed with COVID-19, which is known as a breakthrough SARS-CoV-2 infection (BTI). Al-Aly et al. (30) showed that in long COVID, six months after infection, people with BTI exhibited a higher risk of death and incident post-acute sequelae, including cardiovascular, coagulation, hematologic, gastrointestinal, kidney, mental health, metabolic, musculoskeletal, and neurologic disorders. These results were consistent when compared against the historical and vaccinated controls. Long COVID is a debilitating syndrome that often includes persisting respiratory symptoms and, to a lesser degree, abnormalities in lung physiology (100). Respiratory features of long COVID may decrease over time, yet resolution is not achieved in all cases.

We have previously published that impairments of the electron transport chain and mitochondrial DNA damage increase ROS production, and so-called mitochondria caused oxidative damage (101). COVID-19 might influence mitochondrial function and induce mitochondrial damage, especially in the mitochondrial electron transport chain, and may cause mitochondrial oxidative damage.

Emerging evidence suggests that COVID-19 highjacks mitochondria of immune cells replicates within mitochondrial structures, and impairs mitochondrial dynamics, leading to cell death. Increasing evidence suggests that mitochondria from COVID-19-infected cells are highly vulnerable, and vulnerability increases with age (102). The relationship between long COVID and mitochondria has been focused on. First, after infection of COVID-19, the localization of the virus should be focused. Wu et al. performed computational modeling of SARS-CoV-2 viral RNA localization across eight subcellular organelles: endoplasmic reticulum (ER) membrane, Nuclear lamina, Mito matrix, Cytosol, Nucleolus, Nucleus, Nuclear pore, and Mitochondria outer membrane. We compare hundreds of SARS-CoV-2 genomes to the human transcriptome and other coronaviruses and perform systematic sub-sequence analyses to identify the responsible signals. Using state-of-the-art machine learning models, we predict that the SARS-CoV-2 RNA genome and all sgRNAs are the most enriched in the host mitochondrial matrix (103). Interestingly, Padhaan et al. described that the severe acute respiratory syndrome coronavirus 3a protein activates the mitochondrial death pathway through p38 MAP kinase activation in 2008 (104). Cumpstay proposed the anti-ROS agents as the treatment tool against COVID-19, a redox disease (105). Chen et al. proposed possible pathogenesis and prevention of Long COVID considering SARS-CoV-2-induced mitochondrial disorder (106). Therefore, the most likely COVID-19 goes to mitochondria after the infection into the cells of the host patients and the severe ROS generation from mitochondria that destroys mitochondria and mitochondrial DNA, consequently less oxidative phosphorylation and shift to glycolysis, long COVID symptoms.

**Figure 3:**
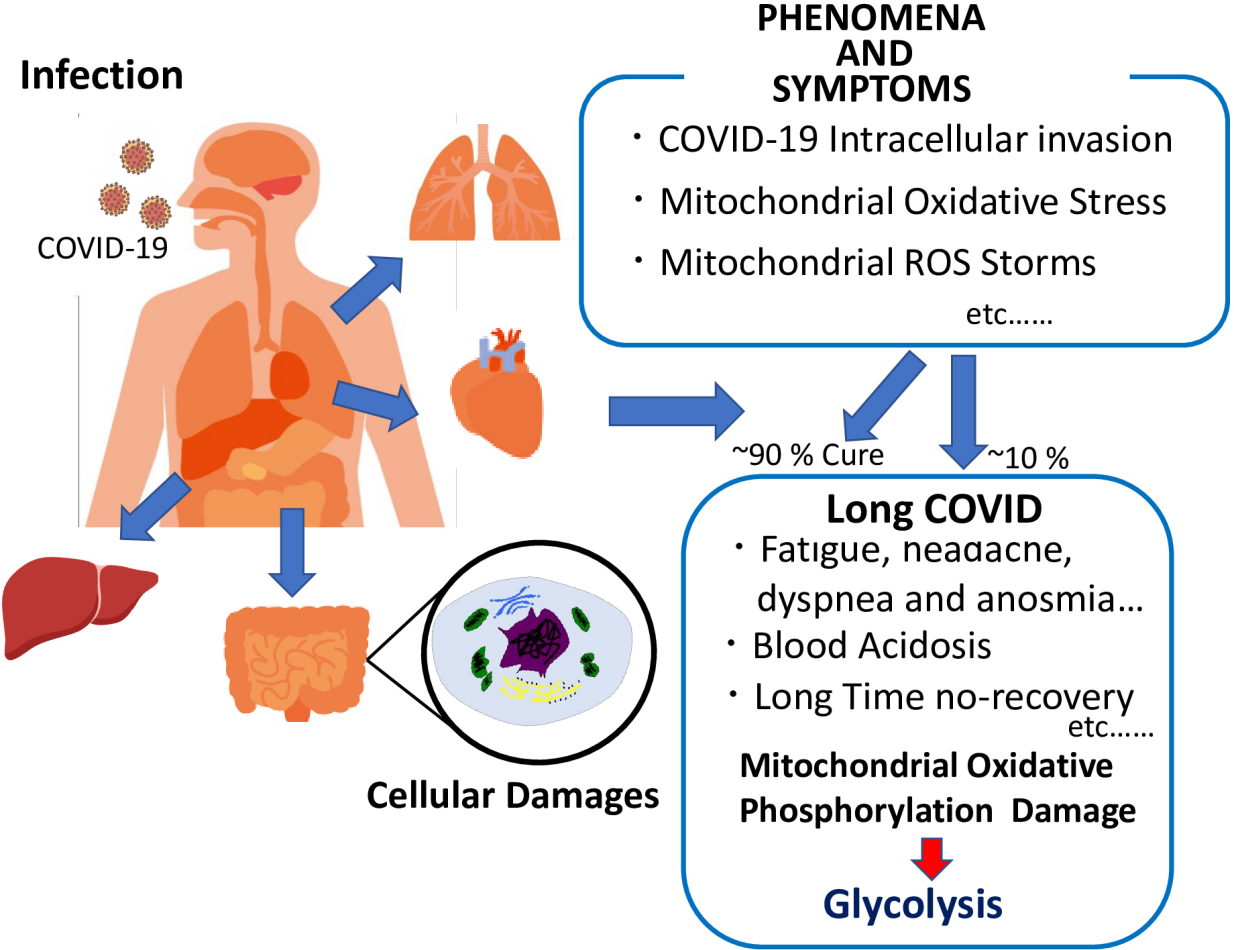
Schematic representation of COVID-19 phenomena and symptoms, and long COVID characteristics.

In conclusion, we summarized the mode of spread, clinical symptoms, infection route, and intracellular signaling of COVID-19, as well as the combination of COVID-19 and diabetes, COVID-19 intracellular invasion, including mitochondrial oxidative stress, mitochondrial ROS storm that destroys mitochondria and electron transport chain (ETC), and causes long COVID (summarized in **Figure 3**). We highlight that the mitochondria might be involved in the pathogenesis of long COVID and symptom manifestation. A comprehensive map of the stages and pathogenetic mechanisms related to the disease and effective drugs to treat and prevent long COVID are urgently required, warranting further investigation on long COVID treatments and symptom relief strategies.

## Author contributions

KN: Investigation, Methodology, Validation, Writing – original draft. MC: Data curation, Validation, Writing – original draft. SiS: Data curation, Investigation, Validation, Writing – original draft. MKo: Data curation, Formal Analysis, Investigation, Validation, Visualization, Writing – review & editing. RH: Methodology, Validation, Writing – original draft. KB: Investigation, Validation, Writing – review & editing. CN: Investigation, Validation, Writing – original draft. AT: Investigation, Validation, Writing – review & editing. WK: Data curation, Investigation, Validation, Writing – original draft. MI: Data curation, Investigation, Validation, Writing – review & editing. FK: Investigation, Validation, Writing – review & editing, Supervision. MKu: Data curation, Investigation, Validation, Writing – review & editing. YK: Data curation, Methodology, Validation, Writing – review & editing. HI: Data curation, Funding acquisition, Methodology, Validation, Writing – review & editing. TK: Data curation, Investigation, Validation, Writing – review & editing. SuS: Data curation, Methodology, Validation, Writing – review & editing. VS: Data curation, Methodology, Validation, Writing – review & editing. LU: Data curation, Methodology, Validation, Writing – review & editing. TI: Data curation, Methodology, Validation, Writing – review & editing. VN: Data curation, Methodology, Validation, Writing – original draft. HI: Methodology, Validation, Writing – review & editing, Conceptualization, Investigation, Supervision, Writing – original draft. JT: Supervision, Writing – review & editing, Data curation. HM: Conceptualization, Data curation, Formal Analysis, Investigation, Methodology, Project administration, Resources, Supervision, Validation, Visualization, Writing – original draft, Writing – review & editing.

## Supplementary material

Supplementary Figure 1 Case mortality ratio (DM vs. others) of patients with COVID-19 and diabetes mellitus (DM) in seven articles (20–26) and the average 1.67.

Supplementary Figure 2 Lactate levels in coronavirus disease 2019 (COVID-19) survivors vs. non-survivors. The forest plot shows significant differences in mean lactate levels between COVID-19 survivors and non-survivors (p < 0.01). Blue-squared box, effect estimate; Green diamond, the pooled effect estimate; Gray vertical line, no effect line; Red vertical line, the pooled effect estimate; N, number of individuals; SD, standard deviation; Mean Diff, mean difference. Remended from Carpenè et al. (2021) (86) and (87–96).

## References

[1] Carvalho T Krammer F Iwasaki A. The first 12 months of COVID-19: a timeline of immunological insights. Nat Rev Immunol (2021) 21:245–56. doi: 10.1038/s41577-021-00522-1 PMC7958099 33723416

[2] Huang C Wang Y Li X Ren L Zhao J Hu Y. Clinical features of patients infected with 2019 novel coronavirus in Wuhan, China. Lancet (2020) 395(10223):497–506. doi: 10.1016/S0140-6736(20)30183-5 31986264 PMC7159299

[3] Chen N Zhou M Dong X Qu J Gong F Han Y. Epidemiological and clinical characteristics of 99 cases of 2019 novel coronavirus pneumonia in Wuhan, China: a descriptive study. Lancet (2020) 395(10223):507–13. doi: 10.1016/S0140-6736(20)30211-7 PMC7135076 32007143

[4] Chan JF Yuan S Kok KH To KK Chu H Yang J. A familial cluster of pneumonia associated with the 2019 novel coronavirus indicating person-to-person transmission: a study of a family cluster. Lancet (2020) 395(10223):514523. doi: 10.1016/S0140-6736(20)30154-9 PMC7159286 31986261

[5] Lu R Zhao X Li J Niu P Yang B Wu H. Genomic characterisation and epidemiology of 2019 novel coronavirus: implications for virus origins and receptor binding. Lancet (2020) 395:565–74. doi: 10.1016/S0140-6736(20)30251-8 PMC7159086 32007145

[6] Yang Y Peng F Wang R Yange M Guan K Jiang T. The deadly coronaviruses: The 2003 SARS pandemic and the 2020 novel coronavirus epidemic in China. J Autoimmun (2020) 109:102434. doi: 10.1016/j.jaut.2020.102434 32143990 PMC7126544

[7] Zhou P Yang XL Wang XG Hu B Zhang L Zhang W. A pneumonia outbreak associated with a new coronavirus of probable bat origin. Nature (2020) 579(7798):270–3. doi: 10.1038/s41586-020-2012-7 PMC7095418 32015507

[8] Seyed Hosseini E Riahi Kashani N Nikzad H Azadbakht J Hassani Bafrani H Hadded Kashani H. The novel coronavirus disease-2019 (COVID-19): mechanism of action, detection and recent therapeutic strategies. Virology (2020) 551:1–9. doi: 10.1016/j.virol.2020.08.011 33010669 PMC7513802

[9] Tang X Wu C Li X Song Y Yao X Wu X. On the origin and continuing evolution of SARS-CoV-2. Natl Sci Rev (2020) 7:1012–23. doi: 10.1093/nsr/nwaa036 PMC7107875 34676127

[10] Zhu N Zhang D Wang W Li X Yang B Song J. A novel coronavirus from patients with pneumonia in China, 2019. N Engl J Med (2020) 382:727–33. doi: 10.1056/NEJMoa2001017 PMC7092803 31978945

[11] Wu F Zhao S Yu B Chen YM Wang W Song ZG. A new coronavirus associated with human respiratory disease in China. Nature (2020) 579(7798):265–9. doi: 10.1038/s41586-020-2008-3 PMC7094943 32015508

[12] Lazarevic I Pravica V Miljanovic D Cupic M. Immune evasion of SARS-CoV-2 emerging variants: what have we learnt so far? Viruses (2021) 13:1192. doi: 10.3390/v13071192 34206453 PMC8310325

[13] Burki TK. Lifting of COVID-19 restrictions in the UK and the Delta variant. Lancet Respir Med (2021) 9:e85. doi: 10.1016/S2213-2600(21)00328-3 34265238 PMC8275031

[14] Noori M Nejadghaderi SA Arshi S Carson-Chahhoud K Ansarin K Kolahi AA. Potency of BNT162b2 and mRNA-1273 vaccine-induced neutralizing antibodies against severe acute respiratory syndrome-CoV-2 variants of concern: A systematic review of *in vitro* studies. Rev Med Virol (2022) 32:e2277. doi: 10.1002/rmv.2277 34286893 PMC8420542

[15] National Institute for Health and Care Excellence. COVID-19 Rapid Guideline: Managing the Long-Term Effects of COVID-19 (2020). NG188. Available at: https://www.nice.org.uk/guidance/ng188 (Accessed 12 December 2022).

[16] Karim SSA Karim QA. Omicron SARS-CoV-2 variant: a new chapter in the COVID-19 pandemic. Lancet (2021) 398:2126–8. doi: 10.1016/S0140-6736(21)02758-6 PMC8640673 34871545

[17] Wang D Hu B Hu C Zhu F Liu X Zhang J. Clinical characteristics of 138 hospitalized patients with 2019 novel coronavirus-infected pneumonia in Wuhan, China. JAMA (2020) 323(11):1061–9. doi: 10.1001/jama.2020.1585 PMC7042881 32031570

[18] Sudre CH Murray B Varsavsky T Graham MS Penfold RS Bowyer RC. Attributes and predictors of long COVID. Nat Med (2021) 27(4):626–31. doi: 10.1038/s41591-021-01292-y PMC7611399 33692530

[19] Centers for Disease Control and Prevention, National Center for Health Statistics. Nearly One in Five American Adults Who Have Had COVID-19 Still Have “Long COVID” (2022). Available at: https://www.cdc.gov/nchs/pressroom/nchs_press_releases/2022/20220622.html.

[20] Hussain S Baxi H Chand Jamali M Nisar N Hussain MS. Burden of diabetes mellitus and its impact on COVID-19 patients: A meta-analysis of real-world evidence. Diabetes Metab Syndr (2020) 14:1595–602. doi: 10.1016/j.dsx.2020.08.014 PMC7439970 32862098

[21] Shang L Shao M Guo Q Shi J Zhao Y Xiaokerati J. Diabetes mellitus is associated with severe infection and mortality in patients with COVID-19: A systematic review and meta-analysis. Arch Med Res (2020) 51:700–9. doi: 10.1016/j.arcmed.2020.07.005 PMC7413048 32811670

[22] Saha S Al-Rifai RH Saha S. Diabetes prevalence and mortality in COVID-19 patients: a systematic review, meta-analysis, and meta-regression. J Diabetes Metab Disord (2021) 20:939–50. doi: 10.1007/s40200-021-00779-2 PMC8012080 33821206

[23] Palaiodimos L Chamorro-Pareja N Karamanis D Li W Zavras PD Chang KM. Diabetes is associated with increased risk for in-hospital mortality in patients with COVID-19: a systematic review and meta-analysis comprising 18,506 patients. Hormones (2021) 20:305–14. doi: 10.1007/s42000-020-00246-2 PMC7595056 33123973

[24] Corona G Pizzocaro A Vena W Rastrelli G Semeraro F Isidori AM. Diabetes is most important cause for mortality in COVID-19 hospitalized patients: Systematic review and meta-analysis. Rev Endocr Metab Disord (2021) 22:275–96. doi: 10.1007/s11154-021-09630-8 PMC7899074 33616801

[25] Gupta P Gupta M KAtoch N Garg K Garg B. A systematic review and meta-analysis of diabetes associated mortality in patients with COVID-19. Int J Endocrinol Metabol (2021) 19:e113220. doi: 10.5812/ijem.113220 PMC8762284 35069750

[26] Ssentongo P Zhang Y Witmer L Chinchilli VM Ba DM. Association of COVID-19 with diabetes: a systematic review and meta-analysis. Sci Rep (2022) 2:20191. doi: 10.1038/s41598-022-24185-7 PMC9684130 36418912

[27] Liu SP Zhang Q Wang W Zhang M Liu C Xiao X. Hyperglycemia is a strong predictor of poor prognosis in COVID-19. Diabetes Res Clin Practi (2020) 167:108338. doi: 10.1016/j.diabres.2020.108338 PMC7377976 32712122

[28] Paul BD Lemle MD Komaroff AL Snyder SH. Redox imbalance links COVID-19 and myalgic encephalomyelitis/chronic fatigue syndrome. Proc Natl Acad Sci U.S.A. (2021) 118:e2024358118. doi: 10.1073/pnas.2024358118 34400495 PMC8403932

[29] Steenblock C Hassanein M Khan EG Yaman M Kamel M Barbir M. Diabetes and COVID-19: short- and long-term consequences. Horm Metab Res (2022) 54:503–9. doi: 10.1055/a-1878-9566 PMC9363150 35724689

[30] Al-Aly Z Bowe B Xie Y. Long COVID after breakthrough SARS-CoV-2 infection. Nat Med (2022) 28:1461–7. doi: 10.1038/s41591-022-01840-0 PMC9307472 35614233

[31] Xie Y Al-Aly Z. Risks and burdens of incident diabetes in long COVID: a cohort study. Lancet Diabetes Endocrinol (2022) 10(5):311–21. doi: 10.1016/S2213-8587(22)00044-4 PMC8937253 35325624

[32] Rizvi AA Kathuria A Al Mahmeed W Al-Rasadi K Al-Alawi K Banach M. Post-COVID syndrome, inflammation, and diabetes. J Diabetes Complications (2022) 36(11):108336. doi: 10.1016/j.jdiacomp.2022.108336 36228563 PMC9534783

[33] Bramante CT Buse JB Liebovitz DM Nicklas JM Puskarich MA Cohen K. Outpatient treatment of COVID-19 and incidence of post-COVID-19 condition over 10 months (COVID-OUT): a multicentre, randomised, quadruple-blind, parallel-group, phase 3 trial. Lancet Infect Dis (2023) 23(10):1119–29. doi: 10.1016/S1473-3099(23)00299-2 PMC11259948 37302406

[34] V’kovski P Kratzel A Steiner S Stalder H Thiel V. Coronavirus biology and replication: implications for SARS-CoV-2. Nat Rev Microbiol (2021) 19(3):155–70. doi: 10.1038/s41579-020-00468-6 PMC7592455 33116300

[35] Rizzi M D’Onghia D Tonello S Minisini R Colangelo D Bellan M. COVID-19 biomarkers at the crossroad between patient stratification and targeted therapy: The role of validated 95. and proposed parameters. Int J Mol Sci (2023) 24(8):7099. doi: 10.3390/ijms2408709995 37108262 PMC10138390

[36] Rizzi M Costanzo M Tonello S Matino E Casciaro FG Croce A. Prognostic markers in Hospitalized COVID-19 patients: The role of IP-10 and C-reactive protein. Dis Markers (2022) 2022:3528312. doi: 10.1155/2022/3528312 PMC8886756 35242241

[37] Rizzi M Matino E Costanzo M Casciaro GF Croce A Rizzi E. Baseline plasma gas6 protein elevation predicts adverse outcomes in hospitalized COVID-19 patients. Dis Markers (2022) 1568352. doi: 10.1155/2022/156835297 PMC9070408 35531477

[38] Apostolo D D’Onghia D Tonello S Minisini R Baricich A Gramaglia C. Decreased Gas6 and sAxl plasma levels are associated with hair loss in COVID-19 survivors. Int J Mol (2023) 24:6257. doi: 10.3390/ijms24076257 PMC10094682 37047229

[39] Tonello S Rizzi M Matino E Costanzo M Casciaro GF Croce A. Baseline plasma Gas6 protein elevation predicts adverse outcomes in hospitalized COVID-19 patients. Dis Markers (2022) 2022:1568352. doi: 10.1155/2022/1568352 35531477 PMC9070408

[40] Chen X Zhao B Qu Y Chen Y Xiong J Feng Y. Detectable serum severe acute respiratory syndrome Coronavirus 2 viral load (RNAemia) Is closely correlated with drastically elevated Interleukin 6 level in critically Ill patients with Coronavirus disease 2019. Clin Infect Dis (2020) 71:1937–42. doi: 10.1093/cid/ciaa449 PMC7184354 32301997

[41] Rizzi M Tonello S Morani F Rizzi E Casciaro GF Matino E. CGRP plasma levels correlate with the clinical evolution and prognosis of hospitalized acute COVID-19 patients. Viruses (2022) 14:2123. doi: 10.3390/v14102123 36298678 PMC9611580

[42] Jackson CB Farzan M Chen B Choe H. Mechanisms of SARS-CoV-2 entry into cells. Nat Rev Mol Cell Biol (2022) 23:3–20. doi: 10.1038/s41580-021-00418-x 34611326 PMC8491763

[43] Loffredo L Violi F. COVID-19 and cardiovascular injury: A role for oxidative stress and antioxidant treatment? Int J Cardiol (2020) 312:136. doi: 10.1016/j.ijcard.2020.04.066 32505331 PMC7833193

[44] Delgado-Roche L Mesta F. Oxidative stress as key player in severe acute respiratory syndrome coronavirus (SARS-coV) infection. Arch Med Res (2020) 51:384–7. doi: 10.1016/j.arcmed.2020.04.019 PMC7190501 32402576

[45] Mallick U. Pathogenesis of coViD19—Miscellaneous mechanisms. In: Cardiovascular Complications of COVID-19. Cham: Springer (2022).

[46] Srinivasan K Pandey AK Livingston A Venkatesh S. Roles of host mitochondria in the development of COVID-19 pathology: Could mitochondria be a potential therapeutic target? Mol BioMed (2021) 2:38. doi: 10.1186/s43556-021-00060-1 34841263 PMC8608434

[47] Singh KK Chaubey G Chen JY Suravajhala P. Decoding SARS-CoV-2 hijacking of host mitochondria in COVID-19 pathogenesis. Am J Physiol Cell Physiol (2020) 1(319):C258–67. doi: 10.1152/ajpcell.00224.2020 PMC7381712 32510973

[48] Anand SK Tikoo SK. Viruses aremodulators of mitochondrial functions. Adv Virol (2013) 2013:738794. doi: 10.1155/2013/738794 24260034 PMC3821892

[49] Claus C Liebert UG. A renewed focus on the interplay between viruses and mitochondrial metabolism. Arch Virol (2014) 159:1267–77. doi: 10.1007/s00705-013-1841-1 24343264

[50] Glingston RS Deb R Kumar S Nagotu S. Organelle dynamics and viral infections: at crossroads. Microbes Infect (2019) 21:20–32. doi: 10.1016/j.micinf.2018.06.002 29953921 PMC7110583

[51] Ajaz S McPhail MJ Singh KK Mujib S Trovato FM Napoli S. Mitochondrial metabolic manipulation by SARS-CoV-2 in peripheral blood mononuclear cells of patients with COVID-19. Am J Physiol Cell Physiol (2021) 320:C57–65. doi: 10.1152/ajpcell.00426.2020 PMC7816428 33151090

[52] Prasun P. COVID-19: A mitochondrial perspective. DNA Cell Biol (2021) 40:713–9. doi: 10.1089/dna.2020.6453 33872068

[53] Ren Z Yu Y Chen C Yang D Ding T Zhu L. The triangle relationship between long noncoding RNA, RIG-I-like receptor signaling pathway, and glycolysis. Front Microbiol (2021) 12:807737. doi: 10.3389/fmicb.2021.807737 34917069 PMC8670088

[54] Zhang W Wang G Xu ZG Tu H Hu F Dai J. Lactate is a natural suppressor of RLR signaling by targeting MAVS. Cell (2019) 178:176–189.e15. doi: 10.1016/j.cell.2019.05.003 31155231 PMC6625351

[55] Wu M Pei Z Long G Chen H Jia Z Xia W. Mitochondrial antiviral signaling protein: a potential therapeutic target in renal disease. Front Immunol (2023) 14:1266461. doi: 10.3389/fimmu.2023.1266461 37901251 PMC10602740

[56] Soriano-Teruel PM García-Laínez G Marco-Salvador M Pardo J Arias M DeFord C. Identification of an ASC oligomerization inhibitor for the treatment of inflammatory diseases. Cell Death Dis (2021) 12:1155. doi: 10.1038/s41419-021-04420-1 34903717 PMC8667020

[57] Valdés-Aguayo JJ Garza-Veloz I Badillo-Almaráz JI Bernal-Silva S Martínez-Vázquez MC Juárez-Alcalá V. Mitochondria and mitochondrial DNA: key elements in the pathogenesis and exacerbation of the inflammatory state caused by COVID-19. Medicina (Kaunas) (2021) 57:928. doi: 10.3390/medicina57090928 34577851 PMC8471487

[58] Kelley N Jeltema D Duan Y He Y. The NLRP3 inflammasome: an overview of mechanisms of activation and regulation. Int J Mol Sci (2019) 20(13):3328. doi: 10.3390/ijms20133328 31284572 PMC6651423

[59] Moreno Fernández-Ayala DJ Navas P López-Lluch G. Age-related mitochondrial dysfunction as a key factor in COVID-19 disease. Exp Gerontol (2020) 142:111147. doi: 10.1016/j.exger.2020.111147 33171276 PMC7648491

[60] Akbari H Taghizadeh-Hesary F. COVID-19 induced liver injury from a new perspective: Mitochondria. Mitochondrion (2023) 70:103–10. doi: 10.1016/j.mito.2023.04.001 PMC10088285 37054906

[61] Prasada Kabekkodu S Chakrabarty S Jayaram P Mallya S Thangaraj K Singh KK. Severe acute respiratory syndrome coronaviruses contributing to mitochondrial dysfunction: Implications for post-COVID complications. Mitochondrion (2023) 69:43–56. doi: 10.1016/j.mito.2023.01.005 36690315 PMC9854144

[62] Guarnieri JW Dybas JM Fazelinia H Kim MS Frere J Zhang Y. Core mitochondrial genes are down-regulated during SARS-CoV-2 infection of rodent and human hosts. Sci Transl Med (2023) 15(708):eabq1533. doi: 10.1126/scitranslmed.abq1533 37556555 PMC11624572

[63] Miller B Silverstein A Flores M Cao K Kumagai H Mehta HH. Host mitochondrial transcriptome response to SARS-CoV-2 in multiple cell models and clinical samples. Sci Rep (2021) 11:3. doi: 10.1038/s41598-020-79552-z 33420163 PMC7794290

[64] Bhowal C Ghosh S Ghatak D De R. Pathophysiological involvement of host mitochondria in SARS-CoV-2 infection that causes COVID-19: a comprehensive evidential insight. Mol Cell Biochem (2023) 478:1325–43. doi: 10.1007/s11010-022-04593-z PMC9617539 36308668

[65] Duan C Ma R Zeng X Chen B Hou D Liu R. SARS-CoV-2 achieves immune escape by destroying mitochondrial quality: Comprehensive analysis of the cellular landscapes of lung and blood specimens from patients with COVID-19. Front Immunol (2022) 13:946731. doi: 10.3389/fimmu.2022.946731 35844544 PMC9283956

[66] Yang Y Wu Y Meng X Wang Z Younis M Liu Y. SARS-CoV-2 membrane protein causes the mitochondrial apoptosis and pulmonary edema via targeting BOK. Cell Death Differ (2022) 29:1395–408. doi: 10.1038/s41418-022-00928-x PMC8752586 35022571

[67] Wu Z McGoogan JM. Characteristics of and important lessons from the coronavirus disease 2019 (COVID-19) outbreak in China: Summary of a Report of 72 314 cases from the Chinese Center for Disease Control and Prevention. JAMA (2020) 323:1239–42. doi: 10.1001/jama.2020.2648 32091533

[68] Raveendran AV Jayadevan R Sashidharan S. Long COVID: an overview. Diabetes Metabo Syndr (2021) 15:869–75. doi: 10.1016/j.dsx.2021.04.007 PMC8056514 33892403

[69] National Center for Immunization and Respiratory Diseases Division of Viral Diseases Centers for Disease Control and Prevention. Long COVID or post-COVID conditions (2022). Available at: https://www.cdc.gov/coronavirus/2019-ncov/long-term-effects/index.html (Accessed 28 November 2022).

[70] Nguyen NN Hoang VT Dao TL Meddeb L Lagier JC Million M. Long-term persistence of symptoms of dyspnoea in COVID-19 patients. Int J Infect Dis (2022) 115:17–23. doi: 10.1016/j.ijid.2021.11.035 34848374 PMC8627107

[71] Crook H Raza S Nowell J Young M Edison P. Long covid-mechanisms, risk factors, and management. BMJ (2021) 374:n1648. doi: 10.1136/bmj.n1648 34312178

[72] Garner P. For 7 weeks I have been through a roller coaster of ill health, extreme emotions, and utter exhaustion (2020). Available at: https://blogs.bmj.com/bmj/2020/05/05/paul-garner-people-who-have-a-more-protracted-illness-need-help-to-understand-and-cope-with-the-constantly-shifting-bizarre-symptoms (Accessed 5 May 2021).

[73] National Institute for Health and Care Excellence. COVID-19 Rapid Guideline: Managing the Long-Term Effects of COVID-19 (2020) . NG188. Available at: https://www.nice.org.uk/guidance/ng188 (Accessed 12 December 2022).

[74] Datta SD Talwar A Lee JT. A proposed framework and timeline of the spectrum of disease due to SARS-CoV-2 infection: illness beyond acute infection and public health implications. JAMA (2020) 324:2251–2. doi: 10.1001/jama.2020.22717 33206133

[75] Davis HE McCorkell L Vogel JM Topol EJ. Long COVID: major findings, mechanisms and recommendations. Nat Rev Microbiol (2023) 21:133–46. doi: 10.1038/s41579-022-00846-2 PMC9839201 36639608

[76] Barrea L Verde L Grant WB Frias-Toral E Sarno G Vetrani C. Vitamin D: A role also in long COVID-19? Nutrients (2022) 14:1625. doi: 10.3390/nu14081625 35458189 PMC9028162

[77] Nalbandian A Sehgal K Gupta A Madhavan MV McGroder C Stevens JS. Post-acute COVID-19 syndrome. Nat Med (2021) 27:601–15. doi: 10.1038/s41591-021-01283-z PMC8893149 33753937

[78] Bateman L Bested AC Bonilla HF Chheda BV Chu L Curtin JM. Myalgic encephalomyelitis/chronic fatigue syndrome: essentials of diagnosis and management. Mayo Clin Proc (2021) 96:2861–78. doi: 10.1016/j.mayocp.2021.07.004 34454716

[79] Wood E Hall KH Tate W. Role of mitochondria, oxidative stress and the response to antioxidants in myalgic encephalomyelitis/chronic fatigue syndrome: A possible approach to SARS-CoV-2 ‘long-haulers’? Chronic Dis Transl Med (2021) 7:14–26. doi: 10.1016/j.cdtm.2020.11.002 33251031 PMC7680046

[80] Azcue N Del Pino R Acera M Fernández-Valle T Ayo-Mentxakatorre N Pérez-Concha T. Dysautonomia and small fiber neuropathy in post-COVID condition and Chronic Fatigue Syndrome. J Transl Med (2023) 21:814. doi: 10.1186/s12967-023-04678-3 37968647 PMC10648633

[81] Linnhoff S Koehler L Haghikia A Zaehle T. The therapeutic potential of non-invasive brain stimulation for the treatment of Long-COVID-related cognitive fatigue. Front Immunol (2023) 13:935614. doi: 10.3389/fimmu.2022.935614 36700201 PMC9869163

[82] Bellan M Apostolo D Albè A Crevola M Errica N Ratano G. Determinants of long COVID among adults hospitalized for SARS-CoV-2 infection: A prospective cohort study. Front Immunol (2022) 13:1038227. doi: 10.3389/fimmu.2022.1038227 36601115 PMC9807078

[83] Nunn AVW Guy GW Brysch W Bell JD. Understanding long COVID; mitochondrial health and adaptation-old pathways, new problems. Biomedicines (2022) 10:3113. doi: 10.3390/biomedicines10123 36551869 PMC9775339

[84] Vollbracht C Kraft K. Oxidative stress and hyper-inflammation as major drivers of severe COVID-19 and long COVID: implications for the benefit of high-dose intravenous vitamin C. Front Pharmacol (2022) 13:899198. doi: 10.3389/fphar.2022.899198 35571085 PMC9100929

[85] López-Hernández Y Monárrez-Espino J López DAG Zheng J Borrego JC Torres-Calzada C. The plasma metabolome of long COVID patients two years after infection. Sci Rep (2023) 1(13):12420. doi: 10.1038/s41598-023-39049-x PMC10394026 37528111

[86] Carpenè G Onorato D Nocini R Fortunato G Rizk JG Henry BM. Blood lactate concentration in COVID-19: a systematic literature review. Clin Chem Lab Med (2021) 60:332–7. doi: 10.1515/cclm-2021-1115 34856090

[87] Wendel Garcia PD Fumeaux T Guerci P Heuberger DM Montomoli J Roche-Campo F. Prognostic factors associated with mortality risk and disease progression in 639 critically ill patients with COVID-19 in Europe: Initial report of the international RISC-19-ICU prospective observational cohort. EClinicalMedicine (2020) 25:100449. doi: 10.1016/j.eclinm.2020.100449 32838231 PMC7338015

[88] Oliveira J Gameiro J Bernardo J Marques F Costa C Branco C. Impact of chronic RAAS use in elderly COVID-19 patients: A retrospective analysis. J Clin Med (2021) 10:3147. doi: 10.3390/jcm10143147 34300311 PMC8307646

[89] Alharthy A Aletreby W Faqihi F Balhamar A Alaklobi F Alanezi K. Clinical characteristics and predictors of 28-day mortality in 352 critically ill patients with COVID-19: A retrospective study. J Epidemiol Glob Health (2021) 11:98–104. doi: 10.2991/jegh.k.200928.001 33095982 PMC7958266

[90] Birben B Birben OD Akın T Akkurt G Surel AA Yakısık E. Efficacy of the delta neutrophil index in predicting 30-day mortality in COVID-19 patients requiring intensive care. Int J Clin Pract (2021) 75:e13970. doi: 10.1111/ijcp.13970 33368905 PMC7883061

[91] Vassiliou AG Jahaj E Ilias I Markaki V Malachias S Vretoo C. Lactate kinetics reflect organ dysfunction and are associated with adverse outcomes in intensive care unit patients with COVID-19 pneumonia: preliminary results from a Greek single-centre study. Metabolites (2020) 10:386. doi: 10.3390/metabo10100386 32998323 PMC7599563

[92] Zhao Y Nie HX Hu K Wu XJ Zhang YT Wang MM. Abnormal immunity of non-survivors with COVID-19: predictors for mortality. Infect Dis Poverty (2020) 9:108. doi: 10.1186/s40249-020-00723-1 32746940 PMC7396941

[93] Sarfaraz S Shaikh Q Saleem SG Rahim A Herekar FF Junejo S. Determinants of in-hospital mortality in COVID-19; a prospective cohort study from Pakistan. PloS One (2021) 16:e0251754. doi: 10.1371/journal.pone.0251754 34043674 PMC8158897

[94] Kalabin A Mani VRK Valdivieso SC Donaldson B. Role of neutrophil-to-lymphocyte, lymphocyte-to-monocyte and platelet-to-lymphocyte ratios as predictors of disease severity in COVID-19 patients. Infez Med (2021) 29:46–53. 33664172

[95] Li R Hu S Chen P Jiang J Cui G Wang DW. Saving critically ill COVID-19 patients with mechanical circulatory support. Ann TranslMed (2021) 9:1221. doi: 10.21037/atm-20-5169 PMC8421987 34532358

[96] Zhang L Li J Zhou M Chen Z. Summary of 20 tracheal intubation by anesthesiologists for patients with severe COVID-19 pneumonia: retrospective case series. J Anesth (2020) 34:599–606. doi: 10.1007/s00540-020-02778-8 32303885 PMC7164839

[97] Martha JW Wibowo A Pranata R. Prognostic value of elevated lactate dehydrogenase in patients with COVID-19: a systematic review and meta-analysis. Postgrad Med J (2022) 98:422–7. doi: 10.1136/postgradmedj-2020-139542 33452143

[98] Iepsen UW Plovsing RR Tjelle K Foss NB Meyhoff CS Ryrsø CK. The role of lactate in sepsis and COVID-19: Perspective from contracting skeletal muscle metabolism. Exp Physiol (2022) 107:665–73. doi: 10.1113/EP089474 PMC8239768 34058787

[99] Antonelli M Pujol JC Spector TD Ourselin S Steves CJ. Risk of long COVID associated with delta versus omicron variants of SARS-CoV-2. Lancet (2022) 399:2263–4. doi: 10.1016/S0140-6736(22)00941-2 PMC9212672 35717982

[100] Daines L Zheng B Pfeffer P Hurst JR Sheikh A. A clinical review of long-COVID with a focus on the respiratory system. Curr Opin Pulm Med (2022) 28:174–9. doi: 10.1097/MCP.0000000000000863 PMC7612723 35131989

[101] Indo HP Davidson M Yen H-C Suenaga S Tomita K Nishii T. Evidence of ROS generation by mitochondria in cells with impaired electron transport chain and mitochondrial DNA damage. Mitochondrion (2007) 7:106–18. doi: 10.1016/j.mito.2006.11.026 17307400

[102] Ganji R Reddy PH. Impact of COVID-19 on mitochondrial-based immunity in aging and age-related diseases. Front Aging Neurosci (2021) 12:614650. doi: 10.3389/fnagi.2020.614650 33510633 PMC7835331

[103] Wu K Zou J Chang HY. RNA-GPS predicts SARS-coV-2 RNA localization to host mitochondria and nucleolus. bioRxiv (2020) 2020:4. doi: 10.1101/2020.04.28.065201 PMC7305881 32673562

[104] Padhan K Minakshi R Towheed MAB Jameel S. Severe acute respiratory syndrome coronavirus 3a protein activates the mitochondrial death pathway through p38 MAP kinase activation. J Gen Virol (2008) 89(Pt 8):1960–9. doi: 10.1099/vir.0.83665-0 18632968

[105] Cumpstey AF Clark AD Santolini J Jackson AA Feelisch M. COVID-19: A redox disease-what a stress pandemic can teach us about resilience and what we may learn from the reactive species interactome about its treatment. Antioxid Redox Signal (2021) 35:1226–68. doi: 10.1089/ars.2021.0017 33985343

[106] Chen TH Chang CJ Hung PH. Possible pathogenesis and prevention of long COVID: SARS-coV-2-induced mitochondrial disorder. Int J Mol Sci (2023) 24:8034. doi: 10.3390/ijms24098034 37175745 PMC10179190

