# Supplementary figures and images for: Mitochondrial oxidative stress, mitochondrial ROS storms in long COVID pathogenesis
Source: Front Immunol. 2023 Dec 22;14:1275001. doi: 10.3389/fimmu.2023.1275001 (PMC10766822; doi:10.3389/fimmu.2023.1275001)

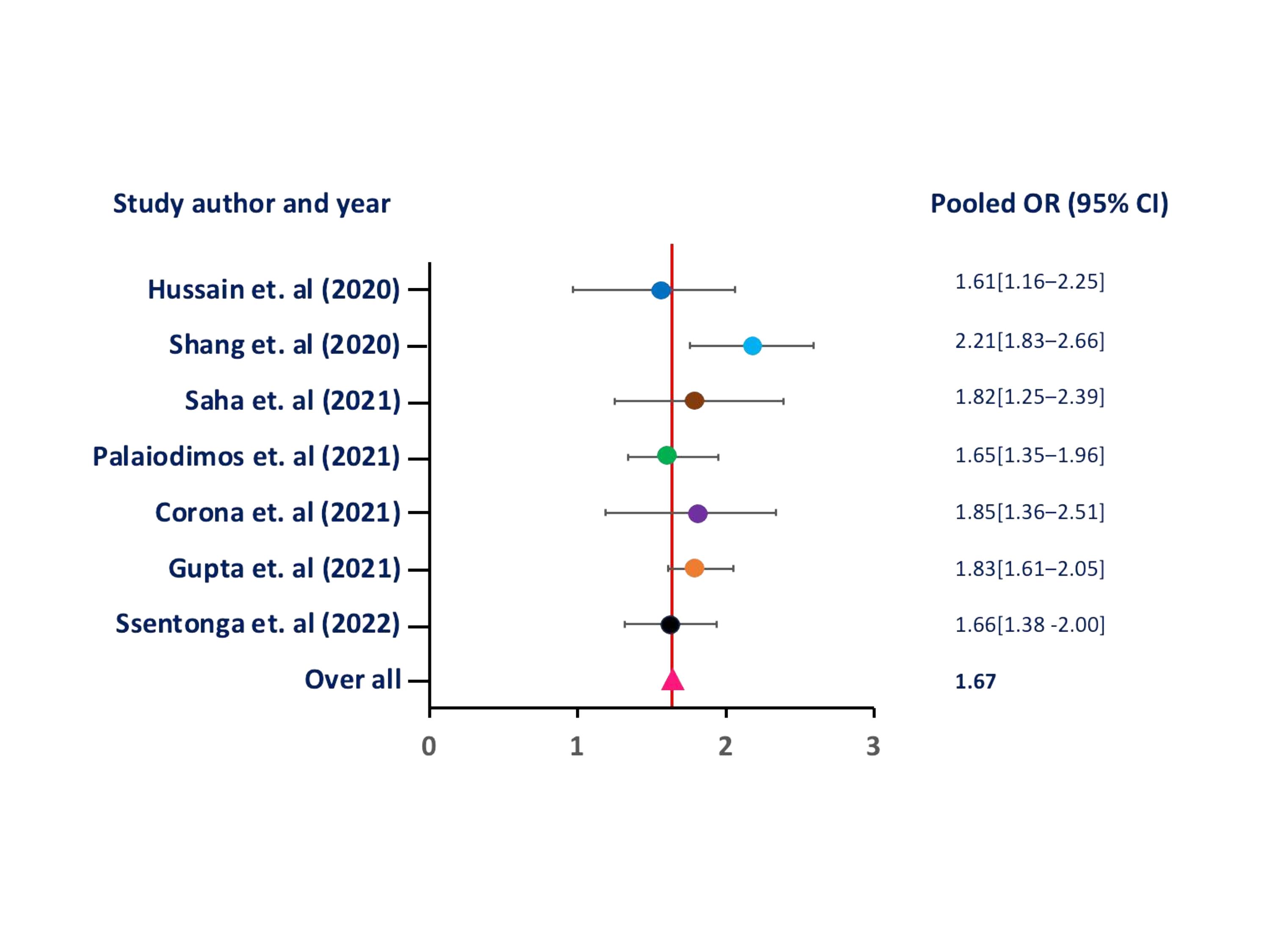

Supplement: Supplementary Figure 1 — Case mortality ratio (DM vs. others) of patients with COVID-19 and diabetes mellitus (DM) in seven articles (20–26) and the average 1.67. [file Image_1.jpeg]

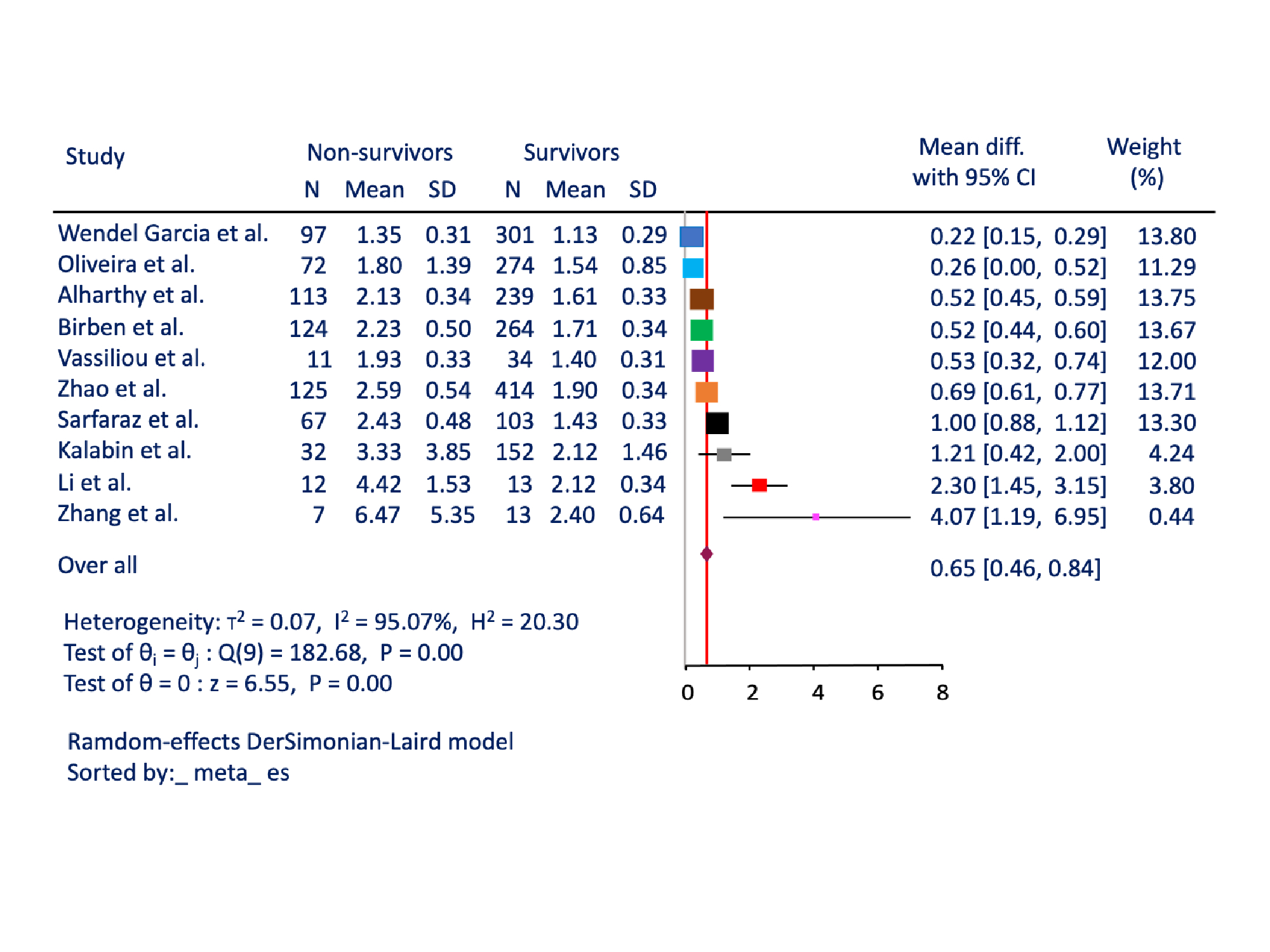

Supplement: Supplementary Figure 2 — Lactate levels in coronavirus disease 2019 (COVID-19) survivors vs. non-survivors. The forest plot shows significant differences in mean lactate levels between COVID-19 survivors and non-survivors (p < 0.01). Blue-squared box, effect estimate; Green diamond, the pooled effect estimate; Gray vertical line, no effect line; Red vertical line, the pooled effect estimate; N, number of individuals; SD, standard deviation; Mean Diff, mean difference. Remended from Carpenè et al. (2021) (86) and (87–96). [file Image_2.jpeg]
